# Barriers and facilitators to accessing post sexual-based violence health services among young women attending higher education institutions in Nigeria

**DOI:** 10.1186/s12905-025-03714-2

**Published:** 2025-04-19

**Authors:** Ajoke Esther Ogedegbe, Zhong Eric Chen, Oluwafemi Adeagbo, Oluwaseun Badru, Ebele R.I. Mogo, Brenda Mbouamba Yankam, Adaeze Oreh, Muktar A. Gadanya, Luchuo Engelbert Bain

**Affiliations:** 1 Adolescents and Youth Wellbeing Initiative, Abuja, FCT Nigeria; 2 Department of Health Behavior and Health Education, College of Public Health, University of Arkansas for Medical Sciences, Little Rock, Arkansas USA; 3 University of Edinburgh, Edinburgh, Scotland UK; 4 Department of Community and Behavioral Health, University of Iowa, Iowa, USA; 5 Department of Sociology, University of Johannesburg, Johannesburg, Auckland Park South Africa; 6 ERIM Consulting, Chicago, USA; 7 University of Cambridge Centre for Human Inspired Artificial Intelligence, Cambridge, England; 8 Fakultät für Mathematik Ruhr-Universität Bochum, Bochum, Germany; 9 Douala General Hospital, Douala, Cameroon; 10 Rivers State Ministry of Health, Port-Harcourt, Rivers State Nigeria; 11 Department of Community Medicine, Bayero University/Aminu Kano Teaching Hospital, Kano, Nigeria; 12 Department of Psychology, Faculty of Humanities, University of Johannesburg, Johannesburg, Auckland Park South Africa; 13 African Population and Health Research Center, Nairobi, Kenya

**Keywords:** Young women, Sexual-based violence, Health services, Higher institutions, Nigeria

## Abstract

**Background:**

Post sexual-based violence (SBV) services are crucial for mitigating SBV-induced consequences. However, these services are reportedly rare and often underutilized, particularly by young women in Sub-Saharan Africa. This study aimed to explore the barriers and facilitators to accessing post-SBV services among young women (18–24 years) attending higher education institutions in Nigeria.

**Methods:**

An online survey, using a piloted questionnaire, was administered to a purposive sample of 114 participants recruited from social media platforms between the 8th and 22nd March 2022. Descriptive statistics were used to summarize the study findings.

**Results:**

The majority (71.1%) of the participants were between the ages of 21 and 24 years. Of the 37 participants who indicated they have had their first sexual intercourse, a quarter (9, 24.3%) indicated it was non-consensual. Also, 1 in 5 respondents did not identify SBV/abuse as abnormal. Half of the participants (50.9%) strongly agreed that a post-SBV health service should be the first place to seek care following an incident of rape, however, over half (53.2%) reported a lack of awareness of existing post-SBV health services as a key barrier affecting access. Less than half of the participants strongly agreed that healthcare workers could provide the post-SBV services highlighted in the study, including emergency contraceptives to prevent pregnancy (42.9%) and post-exposure prophylaxis (PEP) to prevent human immunodeficiency virus (HIV) (39.6%), highlighting awareness gaps. Other significant barriers included stigma, shame, and a lack of support systems. Key facilitators included assurance of confidentiality and access to free post-SBV health services.

**Conclusion:**

Significant barriers and facilitators affect access to post-SBV health services in Nigeria, particularly among young women. Multilevel efforts by families, civil society organizations, communities, and governments are essential to address these barriers and improve access to post-SBV health services.

**Supplementary Information:**

The online version contains supplementary material available at 10.1186/s12905-025-03714-2.

## Introduction

Gender-based violence (GBV) is defined as “violence directed towards a person because of their gender or violence that affects persons of particular genders disproportionately due to structural and societal power imbalances” [1]. The Council of Europe Convention on Preventing and Combating Violence Against Women identifies four major forms of GBV: physical, sexual, emotional/psychological, and economic violence [2]. According to a population study, at least one in three women worldwide has experienced physical, sexual, or other forms of abuse in her lifetime [3]. Violence against women, especially sexual-based violence (SBV) and intimate partner violence (IPV) represents a critical public health issue and a violation of women’s human rights [4]. In Sub-Saharan Africa (SSA), adolescents and young people face high rates of GBV [5], especially SBV during dating and this predominantly occurs in early adulthood [6].

Recognizing the importance of addressing GBV, a Kenyan study found that most GBV survivors were unaware of available GBV services and the need for treatment following such encounters [7]. Similarly, in the Democratic Republic of Congo, only half of the 85% of women who reported being survivors of sexual violence received any post-SBV healthcare services [8]. Mtaita et al. (2021) [9] emphasize the importance of healthcare access for GBV survivors, underscoring the crucial role it plays in reducing the impact of sexual and gender-based violence (SGBV) and preventing future occurrences. Despite this recognized need, underutilization of SBV health services remains widespread across SSA, with some African countries lacking any recorded access to these services [10]. Understanding the barriers and facilitators to accessing post-SBV healthcare services is therefore essential as a public health measure to better support SGBV survivors.

This study explored factors that facilitate or inhibit access to post-SBV health services among young women (18–24 years) attending higher education institutions in Nigeria. It addressed three research questions; (1) What were young women’s levels of knowledge about GBV and SBV health services in Nigeria? (2) What were their perceptions of post-SBV health services in Nigeria? and (3) what were the perceived barriers and facilitators to accessing post-SBV health services in Nigeria?

## Methodology

### Study area

The study was conducted in Nigeria, a West African country comprising of 36 states and a Federal Capital Territory (FCT), further divided into six geo-political zones. Nigeria has the largest population in Africa [11], with over 200 million people [12]; approximately 42.5% of the population is aged 14 or younger, and 19.6% falls within the 15–24 age range [13].

### Study Method and justification

#### Study population

A cross-sectional quantitative survey was conducted among young women aged 18–24 years enrolled in higher education institutions in Nigeria. This age range was chosen because the research targeted undergraduate students in higher institutions.

#### Eligibility criteria

Eligible participants were young women aged 18–24 in their first to final year of undergraduate studies at Nigerian higher education institutions. Postgraduate students were excluded, as most young people aged 15–24, as defined by the United Nations (UN), are undergraduates in Nigeria. The study includes participants who have experienced violence as well as those who have not.

### Data collection

Data was collected using an online questionnaire administered through Jisc online Surveys (formerly BOS), which was live from 8th to 22nd March 2022.

The first page of the questionnaire outlined the study’s purpose, introduced the principal researcher and included two screening questions on age (Are you currently aged 18–24?’) and educational status (Are you currently studying for an undergraduate degree in a higher education institution?’). Consent was also requested on this page. The questionnaire, written in English, took approximately 10 min to complete.

The study’s questionnaire was developed based on questions from three relevant studies conducted in Tanzania and Kenya with similar objectives [7, 9, 15]. The questionnaires from the Tanzania studies were adapted to assess respondent’s knowledge and opinion about GBV health services including barriers and facilitators to access such services, while the study from Kenya was used to develop questions on strategies to improve access to GBV health services.

The questionnaire included the following sections: a screening and consent section, a socio-demographic section with ten questions covering age, marital status, parents’ education level, and monthly allowance (collected as categorical variables). It also assessed participants’ knowledge and awareness of GBV and its health impacts, evaluated their understanding and perceptions of SBV health services in Nigeria, and explored barriers and facilitators to accessing these services.

An initial draft of the questionnaire was piloted with five participants to refine and inform the final version. While no changes were made to the questions or response options, the introductory information was revised to improve clarity.

#### Sampling and recruitment

A purposive sampling method was employed in this study, considering the limited time and resources available for conducting a postgraduate project. The limited time for data collection was a factor responsible for the study’s target sample size of 160. This sample size was informed by a systematic review [6] of similar studies that had sample sizes of female participants ranging from 100 to 300.

Participants were recruited by sharing survey links on social media platforms, including Facebook, Instagram, LinkedIn, and Twitter where young people are typically active. The questionnaire was promoted weekly on these platforms. Ensuring representation from all six zones of Nigeria posed an anticipated challenge which was addressed by sharing the survey link through the corresponding author’s contacts, following referrals, and engaging with representatives from student, academic and religious groups within higher institutions nationwide.

### Measures

Knowledge and awareness of GBV and SBV health services were assessed by counting participants’ responses (Yes, No, or Not Sure). Awareness of the four forms of GBV (physical, sexual, emotional, economic), as identified by The Council of Europe Convention on Preventing and Combating Violence Against Women [2], was determined by counting the number of participants who correctly identified each form.

Perceptions of SBV health services were measured on a five-point Likert scale (strongly agree to strongly disagree). Additional analyses focused on barriers and facilitators to SBV health service utilization. The barriers and facilitators highlighted in this study were identified from the literature. These were also rated on a five-point Likert scale. For each barrier and facilitator, counts and percentages were reported.

### Data analysis

Quantitative data was collected and analysed using Jisc Online Surveys (formerly BOS) statistical software, with counts and percentages reported for each response. Missing responses were handled by analyzing available data independently for each question, with the total (n) indicated per question. The results section presents a detailed breakdown of participant feedback and data analysis outcomes.

### Ethical considerations

The research was conducted in accordance with the standard of the Usher Masters Research Ethics Group (UMREG) and the Academic and Clinical Central Office for Research and Development (ACCORD) of the University of Edinburgh, United Kingdom. Ethics approval was also obtained from the FCT Health Research Ethics Committee in Nigeria on the 14th of December 2021. The ACCORD sponsorship number is AC21183, and the Approval number for the FCT Health Research Ethics Committee in Nigeria is FHREC/2021/01/155/14-12-21.

## Results

There are a total of 114 participants in the study. Response rates varied across different questions. Results for the socio-demographic, knowledge/awareness, and perception sections are presented.

### Socio-demographic characteristics of the study participants

Participants represented all six geo-political zones of Nigeria with the majority from South-Western (28.4%), North-Central, (27.4%), and South-Eastern, (23.9%) (Table 1). Most participants were single (91.2%) and the majority (59.3%) reported a monthly allowance above 10,000 Naira, with 76.8% stating that their allowance came from their parents. Regarding age at first intercourse, 39 participants indicated having had first sexual intercourse; 20.2% was between 16 and 20 years, 13.2% between 21 and 24 years and 1 (0.9%) reported first intercourse before age 16. Three quarters of these participants (75.7%) reported that their first intercourse was consensual with almost a quarter (24.3%) indicating it was non-consensual; 2 (5.1%) participants did not provide a response (Table 1).

**Table 1:** Socio-demographic characteristics of participants (*N* = 114)

Characteristics	Frequency *n* (%)		Characteristics	Frequency *n* (%)	
**Age** (***n* = 114)**			Parental level of education* **Father** (***n* = 112)**		
18–20	33 (28.9)				
21–24	81 (71.1)		No formal education	0	
			Secondary education	16 (14.3)	
**State of residence** (***n* = 113)**			Higher education	82 (73.2)	
South-Western Nigeria	32 (28.4)		I don’t know	3 (2.7)	
North-Central Nigeria	31 (27.4)		Not applicable	2 (1.8)	
South-Eastern Nigeria	27 (23.9)				
South-South Nigeria	18 (15.9)		**Mother** (***n* = 113)**		
North-Eastern Nigeria	4 (3.5)		No formal education	1 (0.9)	
North-Western Nigeria	1 (0.9)		Primary education	3 (2.7)	
			Secondary education	22 (19.4)	
**Marital Status** (***n* = 114)**			Higher education	85 (75.2)	
Single	104 (91.2)		I don’t know	1 (0.9)	
Partnered but not cohabiting	10 (8.8)		Not applicable	1 (0.9)	
Cohabiting	0				
Married	0		**Monthly allowance** (***n* = 113)**		
			None	14 (12.4)	
**Age of first intercourse **(***n* = 112)**			Below N5,000	8 (7.1)	
Less than 16 years	1 (0.9)		N5,000-N10,000	24 (21.2)	
16–20 years	23 (20.2)		Above N10,000	67 (59.3)	
21–24 years	15 (13.2)				
None of the above	75 (65.7)		**Monthly allowance source** (***n* = 112)**		
			Parents	86 (76.8)	
**Nature of 1st intercourse** (*n* = 37)*			Other family member	4 (3.6)	
Consensual	28 (75.7)		Intimate partner	1 (0.9)	
Not consensual	9 (24.3)		Part-time job	11 (9.8)	
			Other	10 (8.9)	

* Include only data from participants who indicated they have had first sexual intercourse

### What are young women’s level of knowledge and awareness of GBV and post SBV health services in Nigeria?

#### Participants’ knowledge of the forms of GBV

Respondents were asked to identify violence that can be experienced in an intimate partner relationship. 89% of the 114 participants identified physical and sexual violence as an experience of GBV by an intimate partner. 78% of the participants identified emotional violence as an experience of GBV and just over a third (39.4%) identified economic violence as a form of GBV (Table 2).

**Table 2:** Breakdown of participants’ (*N* = 114) knowledge of gender-based violence (%)

Forms of gender-based violence	Percentage (*n*, percentage)*
Physical	101, 89%
Sexual	101, 89%
Emotional	89, 78%
Economic	45, 39.4%

* Participant who identified (Yes’) the form of violence as gender-based violence

#### Knowledge/awareness of SBV and its health impacts

In response to whether preventing SBV reduces HIV exposure, 72.6% of participants agreed, 11.5% disagreed, and 15.9% were uncertain. Furthermore, 94.7% believed that SBV could result in unintended pregnancies, while 2.6% either disagreed or were unsure (see Table 3).

**Table 3:** Knowledge and awareness of SBV and post SBV health services among the study participants (*N* = 114)

Survey questions	Knowledge and Awareness		
	Yes	No	Not Sure
**Preventing SBV prevents HIV exposure** (***n* = 113)**	82 (72.6%)	13 (11.5%)	18 (15.9%)
**SBV can lead to unintended pregnancies?** (***n* = 114)**	108 (94.7%)	3 (2.6%)	3 (2.6%)
**Indicate if you are aware of the following**			
Contraceptives (*n* = 110)	105 (95.5%)	3 (2.7%)	2 (1.8%)
Emergency contraception (*n* = 105)	70 (66.7%)	31 (29.5%)	4 (3.8%)
Condoms (*n* = 109)	107 (98.2%)	2 (1.8%)	0
PEP for HIV (*n* = 108)	47 (43.5%)	46 (42.6%)	15 (13.9%)
**Indicate if you have used any of the following**			
Contraceptives (*n* = 107)	26 (24.3%)	80 (74.8%)	1 (0.9%)
Emergency contraception (*n* = 105)	23 (21.9%)	81 (77.1%)	1 (1%)
Condom (*n* = 107)	33 (30.8%)	74 (69.2%)	0
PEP for HIV (*n* = 102)	2 (2.0%)	96 (94.1%)	4 (3.9%)
**Have you ever been concerned or worried about your health and well-being while or after having sex with a partner** (*n* = 39)*	30 (76.9%)	8 (20.5%)	1 (2.6%)
If yes, have you spoke to anyone about it (*n* = 30)**	14 (46.7%)	16 (53.3%)	
If yes, have you got support from a SBV-health service (*n* = 30)**	6 (20%)	24 (80%)	

HIV: Human Immunodeficiency Virus; PEP: Post exposure prophylaxis; SBV: sexual-based violence

* Include only data from participants who indicated they have had first sexual intercourse

** Include only data from participants who indicated ‘yes’ in previous question

#### Awareness of strategies/services offered by a post SBV health service

##### Contraception

95.5% (*n* = 110) of the participants reported being aware of contraceptives. 26 participants reported having used contraceptives, representing 24.3% of all participants who answered this question or 24.8% of participants who were aware of contraceptives.

##### Emergency contraception

66.7% of the participants reported being aware of emergency contraception and 29.5% of participants reported they were unaware. 23 participants reported having used emergency contraception, representing 21.9% of all participants who answered this question or 32.9% of participants who were aware of emergency contraception.

##### Condoms

98.2% of participants reported being aware of condoms. 33 participants reported having used condoms, representing 30.8% of all participants who answered this question or 30.8% of participants who were aware of condoms.

##### Post-exposure prophylaxis (PEP) for HIV

43.5% of participants reported being aware of PEP for HIV, with about an equal proportion (42.6%,) reporting they are unaware and the remaining 13.9% unsure. 2 participants reported having used PEP for HIV, representing 2% of all participants who answered this question or 1.9% of participants who were aware of PEP for HIV.

##### Concern about their health

Of the 39 participants who indicated they have had first sexual intercourse, more than three quarters (76.9%) reported they had been worried or concerned about their health during or after having sex with a partner. Of these 30 participants, 14 (46.7%) reported having sought support or spoken to someone about it, and 6 (20%) sought support from a SBV health service facility. (See Table 3)

### What are young women’s perceptions of post SBV health services in Nigeria?

More than 80% of participants affirmed that post-SBV health services should be the first point of contact following rape, with 29.5% agreeing and 50.9% strongly agreeing. Over half of the participants (57.1%) believed that healthcare workers (HCWs) could help prevent SBV from recurring, with 33.9% agreeing and 23.2% strongly agreeing. Additionally, the majority (78.3%) of participants recognized the availability of HCWs to provide post-exposure prophylaxis (PEP) for HIV prevention after rape, with 39.6% strongly agreeing and 38.7% agreeing (see Table 4).

Most participants (90.2%) affirmed that healthcare workers (HCWs) can provide linkages to psychosocial and counselling services for survivors of sexual and gender-based violence (SBV), with 42.0% agreeing and 48.2% strongly agreeing. Similarly, 93.8% of participants indicated that HCWs could facilitate access to legal aid services, with 49.6% agreeing and 44.2% strongly agreeing. When asked about the role of HCWs in evidence collection for forensic medical services (e.g., semen, blood, and hair samples), 92.9% of participants expressed agreement, with 46.9% agreeing and 46.0% strongly agreeing. However, fewer participants (84.9%) supported the idea that SBV survivors should access post-incident health services for treating physical injuries, with 40.7% agreeing and 44.2% strongly agreeing (see Table 4).

**Table 4:** Perceptions of post SBV health services among the study participants (*N* = 114)

Statement	*n*	Agreement (*n*, %)					
		Strongly agree	Agree	Neutral	Disagree	Strongly disagree	
A post SBV health service should be the first place to go following rape	112	57 (50.9)	33 (29.5)	17 (15.2)	4 (3.6)	1 (0.9)	
HCWs can prevent SBV from recurring	112	26 (23.2)	38 (33.9)	35 (31.3)	11 (9.8)	2 (1.8)	
HCW are available to offer PEP for the prevention of HIV following rape	111	44 (39.6)	43 (38.7	18 (16.2)	5 (4.5)	1 (0.9)	
Post SBV health services are effective in offering services to prevent pregnancy following rape	112	48 (42.9)	48 (42.9)	14 (12.5)	1 (0.9)	1 (0.9)	
HCWs can offer or provide linkages for psychological and counseling services to SBV survivors	112	54 (48.2)	47 (42)	10 (8.9)	1 (0.9)	0	
HCWs can link SBV survivors to legal AID services	113	50 (44.2)	56 (49.6)	5 (4.4)	2 (1.8)	0	
HCWs can be helpful in the collection of evidence for forensic medical services	113	52 (46)	53 (46.9)	7 (6.2)	1 (0.9)	0	
SBV survivors should visit post SBV health services to treat physical injuries	113	50 (44.2)	46 (40.7)	14 (12.4)	2 (1.8)	1 (0.9)	

HIV: Human immunodeficiency virus; HCW: Health care workers; PEP: Post exposure prophylaxis; SBV-Sexual-based violence

### What are the barriers and facilitators to accessing post-SBV health services in Nigeria

#### Barriers

A scale from 1 to 5 (with 1 being “not at all” and 5 being “very much”) was used to assess the extent to which nine barriers, identified from the literature, impact young women’s access to post-SBV health services (Table 5). Most participants rated “stigma/shame” (64.7%), “lack of support systems in the home, school, and community to facilitate visits to post-SBV health services” (54.9%), and “lack of awareness of existing post-SBV health services” (54.0%) as significant barriers (“very much”) impacting young women’s access to these services.

The next three factors that just under half of participants rated as significant barriers (“very much”) to young women’s access to post-SBV health services were “financial/cost of accessing services” (50.5%), “fear of reporting the offender” (50.0%), and “negative and unfriendly attitudes, including distrust of healthcare providers” (37.7%).

Around a third or fewer participants rated the final three factors as significant barriers (“very much”) to young women’s access to post-SBV health services: “fear of HIV testing” (37.7%), “difficulty accessing post-SBV health services due to the distance of the facility” (33.3%), and “belief that violence is normal” (25.4%). A minority of participants rated the first two factors as “not at all” important barriers (13.2% for “fear of HIV testing” and 10.6% for “difficulty accessing services due to facility distance”). Notably, one out of five participants indicated the “belief that abuse was normal,” was “not at all” important.

**Table 5:** Participants’ (*N* = 114) rating of barriers to accessing post-SBV health services (%)

Barriers	*n*	Rating (%); 1 = Not at all, 5 = Very much				
		1	2	3	4	5
Stigma/shame	102	3.9	10.8	3.9	16.7	64.7
Lack of support system in the home/school/community	113	12.4	8.8	13.3	10.6	54.9
Lack of awareness of existing post-GBV services	113	10.6	8.8	15.0	11.5	54.0
Financial barrier	93	0	0	24.7	24.7	50.5
Fear of reporting the offender	94	0	0	23.4	26.6	50.0
Negative/ unfriendly attitude/ distrust of service providers	114	9.6	7.9	24.6	20.2	37.7
Fear of HIV testing	114	13.2	6.1	27.2	20.2	33.3
Difficulty in accessing services (distance)	113	10.6	11.5	29.2	15.9	32.7
Belief that the abuse was normal	114	20.2%	10.5%	21.9%	21.9%	25.4%

#### Facilitators

A scale from 1 to 5 (with 1 being “not at all” and 5 being “very much”) was used to assess the extent to which six facilitators, identified from the literature, impact young women’s access to post-SBV health services (Table 6). Most participants rated each of the six suggested facilitators as significant (“very much”) in supporting young women’s access to post-SBV health services. The most important facilitator was “assurance of confidentiality after visiting post-SBV health services” (61.9%), while the least, though still highly rated, was “support systems in homes, schools, and communities to facilitate access to post SBV health services” (53.5%).

**Table 6:** Participant’s (*N* = 114) rating of facilitators to accessing post-SBV health services (%)

Facilitators	*n*	Rating (%); 1 = Not at all, 5 = Very much				
		1	2	3	4	5
Assurance of confidentiality after visiting post SBV health services	113	11.5%	2.7%	13.3%	10.6%	61.9%
Presence of Post SBV health service facilities in the community people live in, e.g., Universities	113	7.1%	3.5%	14.2%	16.8%	58.4%
Free/Affordable Post-SBV health services	113	4.4%	10.6%	15.9%	12.4%	56.6%
Effective care and support from post SBV health services and health workers	113	11.5%	1.8%	15.0%	16.8%	54.9%
Availability of post SBV health services as part of the larger general healthcare services	112	8.9%	6.3%	11.6%	19.6%	53.6%
Support systems in homes, schools, and communities to facilitate access to post SBV health services	114	9.6%	2.6%	18.4%	15.8%	53.5%

## Discussion

This study examined factors influencing access to post-SBV health services among young women aged 18–24 in Nigerian higher education. The discussion addresses the three research questions on knowledge of GBV and SBV health services, perceptions of post-SBV services, and key barriers and facilitators to access. Findings are contextualized within the broader literature, with implications for public health policy and potential targeted interventions to improve service accessibility for young women in Nigeria.

### Level of knowledge of GBV and SBV health services among young women in Nigeria

The study participants demonstrated a high level of knowledge about GBV, with 89% identifying physical and sexual violence and 78% recognizing emotional violence as forms of GBV. This awareness is likely influenced by their education level, as all participants are enrolled in a higher education. Supporting this finding, is a study that show a statistically significant association between the knowledge of GBV and level of education in the South-South region of Nigeria [16], and a Philippines study that show university students are extremely aware of all the forms of GBV [17]. Additionally, most participants reported that their parents had higher education, aligning with findings from SSA that associated higher education and household economic factors with greater GBV awareness [18].

While most young women identified sexual, physical, and emotional abuse as forms of GBV, 39.4% recognized economic violence as a form of GBV. This highlights the need for increased awareness and education among young women in Nigeria to close knowledge gaps regarding the various forms and definition of GBV.

The young women demonstrated awareness that preventing SBV is linked to reducing HIV risk and unintended pregnancies and showed high knowledge of contraceptives and condoms. However, fewer young women were aware of emergency contraception, and less than half knew about PEP for HIV prevention. This limited awareness may be linked to the fact that most surveyed young women were not currently sexually active, leading to lower consideration of their pregnancy and HIV risk and, consequently, limited awareness of these preventive measures. These gaps in knowledge about SBV health services could impact the utilization of these services, potentially leading to poorer outcomes for SBV survivors and negative mental and physical health outcomes [15].

### Perception of post SBV health services

The young women generally perceived SBV health services as the first point of care following sexual abuse, with most agreeing with this view while very few disagreed. This aligns with their strong knowledge of SBV’s reproductive health consequences, such as unintended pregnancies and HIV risk. However, awareness of specific services like emergency contraceptives and PEP for HIV prevention was low, potentially impacting their perception of available SBV health services. The study also revealed limited confidence among participants regarding healthcare providers’ ability to offer essential post-SBV services such as PEP to prevent HIV, emergency contraception, and forensic services. Less than half believed that SBV health services should be sought for treating physical injuries. These findings suggest a need for greater awareness of post-SBV health services, which could improve young women’s perception and willingness to seek these services promptly following SBV incidents or rape [7].

Although the young women generally demonstrated a poor perception of the statement that HCWs can prevent SBV from reoccurring, a notable proportion agreed that HCWs can provide linkages to psychological counseling and legal aid services. This partially supports the idea that HCWs can play a role in preventing SBV recurrence [19]. Future studies may benefit from presenting this statement more clearly. Overall, however, participants showed limited confidence in HCWs’ ability to provide referrals to psychological and legal aid services, which could be instrumental in preventing repeated violence.

### Barriers and facilitators in accessing post-SBV health services

#### Barriers

Stigma and shame were identified as the primary barrier to accessing post-SBV health services. In Nigeria, family and community members may perpetuate stigma, particularly towards young, unmarried women, who are often seen as promiscuous when they report sexual abuse [9]. Societal norms about the ‘appropriate’ age for sexual activities contribute to negative response towards SBV survivors. HCWs, civil society organizations, project implementers, and schools play a crucial role in addressing this stigma and shame [20]. These stakeholders can educate and sensitize communities about SGBV, emphasizing the importance of supporting survivors to speak up and seek help. Further, stigma can also be perpetuated by HCWs themselves, highlighting the need for targeted training to ensure consistent quality care at post-SBV services nationwide as a public health response.

The second most significant barrier to young women’s use of post-SBV health services was the lack of a support system at home, school, and within their community. A qualitative study in Tanzania supports this finding that 13 out of 20 participants relied on immediate family for emotional support, hospital accompaniment and financial assistance following SBV [9]. Community members also played an essential role when needed [9]. This underscores the importance of educating and sensitizing families, schools, and communities about their vital roles in supporting SBV survivors. Without this awareness, families and communities may respond inadequately to SBV cases, negatively affecting survivors’ emotional well-being and willingness to seek help.

The lack of awareness of existing SBV health services was the third most significant barrier to seeking post-SBV care. This aligns with a Kenyan study showing that most SBV survivors were unaware of available SBV recovery services [7]. Increasing awareness requires action from stakeholders such as civil society organizations, schools, HCWs, and government bodies, includes the Ministry of Women Affairs and the National Agency for the Control of AIDS. Awareness efforts could leverage effective channels like radio jingles, television adverts, and other outreach vulnerable groups, especially young women.

#### Facilitators

One of the primary facilitators identified by young women for accessing post-SBV health services was the assurance of confidentiality, which was the highest-rated facilitator in this study and aligns with the findings from the previously mentioned Kenyan study [7]. Ensuring confidentiality can be strengthened through capacity building and consistent and quality training for post-SBV healthcare providers, promoting quality and equitable care nationwide. Training should emphasize making SBV services survivor-friendly and maintaining confidentiality, as a breach of trust in this area can deter other survivors from seeking care [7]. This is consistent with a previous study that shows poor treatment of SBV survivors for example, failure to maintain confidentiality, negatively impacts post SBV health service utilization [21].

The second highest-rated facilitator was the presence of SBV health facilities within the community where people live. Expanding the distribution and availability of accredited SBV health providers can help address gaps in service access. Building the capacity of HCWs to offer specialized SBV health services, particularly in areas with high populations of young women, such as university campuses, could enhance access and encourage service utilization [7]. Increasing SBV healthcare coverage is likely to improve service uptake among those who need it.

Free or affordable GBV-health services were the third highest-rated facilitator for accessing SBV health services with participants identifying this as a crucial factor. This aligns with a Nigerian study showing that AGYW often face financial barriers limiting their access to these services [22]. Access can be improved by offering SBV health services at no or low cost, particularly benefiting young women who are enrolled in school, financially dependent on others or from low-income backgrounds.

## Strengths and limitations of the study

This study provides valuable insight into the knowledge and awareness levels of GBV and post-SBV health services among young women in Nigeria, highlighting existing awareness gaps. It also identifies key barriers and facilitators to accessing these services, contributes to the body of knowledge and potentially informing public health policies and interventions. However, the study has limitations. A purposive sampling method was used, which, while suitable considering the time and resource constraints, may have led to a sample with similar characteristics, affecting the generalizability of the findings. The limited timeframe means that the recruitment may not have disseminated as far as it was intended to recruit more participants.

Another limitation of this study is its reliance on an online survey, potentially excluding eligible young women without internet access. Additionally, young women who are out of school were not included, which may limit the generalizability of the findings. Further, women who have experienced violence may be less likely to respond due to the stigma they may feel, making who have chosen to respond an inherent limitation. It is important to highlight that the women recruited for the study were women 18–24 years and women who were older but may have experienced violence at the target age were not included. In addition, the sample size of 114 may not fully represent the estimated number of students enrolled, which could impact the result’s generalizability. However, this study is intended to serve as a foundation for a more comprehensive future study. Although responses were obtained from all six zones in Nigeria, only one participant was from the North-West and four from the North-East. Furthermore, data collection was limited to a three-week period due to delays and time constraints.

## Conclusion

The study highlighted several barriers and facilitators affecting access to post-SBV health services. Key barriers included a lack of awareness of available post-SBV services and feeling shame or stigma. Facilitators identified included the assurance of confidentiality and the availability of free or affordable SBV health services. While young women demonstrated a good level of knowledge about GBV and its forms, awareness of the specific services offered by SBV health service was limited. Efforts to improve access to post-SBV health services in Nigeria amongst young women should focus on increased awareness of GBV and information about relevant support services. At a more strategic level, to address critical barriers such as stigma, national and regional policies should support targeted training for healthcare workers, aiming to enhance SBV care delivery, minimize stigma within healthcare settings, and improve the overall service experience for SBV survivors seeking support at challenging times.

Key recommendations include investing in education and awareness initiatives to inform individuals and communities about GBV, the available support services, and how to access them. Future research is encouraged to include young girls under 18, out-of-school young women, and those without internet access to broaden understanding in this area. Enhancing knowledge of SBV services is critical to mitigating risks such as HIV, unintended pregnancies, and other adverse outcomes, aligning with Sustainable Development Goal 3: Good Health and Well-being.

## Electronic supplementary material

Below is the link to the electronic supplementary material.

Supplementary Material 1

Supplementary Material 2

## Data Availability

Data was collected and analysed using Jisc online Surveys (formerly BOS) statistical software. This statistical software was provided by the University of Edinburgh, United Kingdom. The analysis involved reporting counts and percentages of responses for each question. Below is the link of the survey.https://edinburgh.onlinesurveys.ac.uk/gbv-health-services-pilot-survey-copy.

## Abbreviations

SBV: Sexual-Based Violence
GBV: Gender-Based Violence
SSA: Sub-Saharan Africa
AGYW: Adolescent Girls and Young Women
HIV: Human Immunodeficiency virus
HCWs: Health Care Workers
PEP: Post Exposure Prophylaxis
UMREG: Usher Masters Research Ethics Group
ACCORD: Academic and Clinical Central Office for Research and Development
