# Supplementary material for: Barriers and facilitators to accessing post sexual-based violence health services among young women attending higher education institutions in Nigeria
Source: BMC Womens Health. 2025 Apr 19;25:193. doi: 10.1186/s12905-025-03714-2 (PMC12008884; doi:10.1186/s12905-025-03714-2)
Supplement: Supplementary file 1 — Supplementary Material 1 [file 12905_2025_3714_MOESM1_ESM.doc]

**ONLINE QUESTIONNAIRE/SURVEY**

**Research Title:**

*Barriers and Facilitators to Accessing Post Sexual-Based Violence (SBV) Health Services among Young Women (18-24 years) attending Higher Education Institutions in Nigeria*

**Introduction:**

Thank you for reading the associated Participant Information Sheet and Consent Form. If you would like to ask any questions about the study, please contact the researcher, Ajoke Adebisi, using the contact details provided on the Participant Information Sheet.

Before completing the questionnaire below, I would like to remind you that your participation is voluntary and you can withdraw at any time from the study without giving reason. The questionnaire will take approximately 10 minutes of your time to complete.

All the information you give in this questionnaire will be kept private and there are strict laws which safeguard your privacy at every stage. You can withdraw from the study at any point before submission of the questionnaire by exiting the survey, and there is no penalty for this. For participants who complete and submit the questionnaire, it may not be possible for them to withdraw from the study because data will be anonymous at the point of submission, and it will be impossible for their responses to be traced.

The results of this research will be written up in a University dissertation and will not contain any information that could personally identify you.

By completing the questionnaire below, you are not only providing your consent to take part in this study but also expressing your understanding of the consent points provided in the Consent Form already provided to you.

**Consent**

Do you give consent to take part in this study? Yes ( ) No ( )

**Questionnaire for this study:**

Please answer the following questions as honestly as possible to the best of your ability to remember. The information you provide in this study will remain confidential. Thank you!

**Socio-demographic Characteristics**

- Age

18-20 ( ) 21-24 ( )

- Place of residence

On campus ( ) off campus ( )

- Living arrangement at home

I have my own room ( ) I share my room with people ( )

- Location of school

North Central Nigeria ( ) South-western Nigeria ( ) South-Eastern Nigeria ( ) North-Eastern Nigeria ( ) North-Western Nigeria ( ) South-South Nigeria ( )

- Marital status

Single ( ) married ( ) cohabiting ( ) Have a partner but not cohabiting ( )

- Level of Parents/guardian education

For Father: No formal education ( ) Primary education ( ) Secondary education ( ) Higher education ( )

For Mother: No formal education ( ) Primary education ( ) Secondary education ( ) Higher education ( )

For guardian: No formal education ( ) Primary education ( ) Secondary education ( ) Higher education ( )

- Primary occupation of parents/guardian

For Father: Employed ( ) Self-employed ( ) Unemployed ( )

For Mother: Employed ( ) Self-employed ( ) Unemployed ( )

For guardian: Employed ( ) Self-employed ( ) Unemployed ( )

- Monthly allowance

Below N5,000 ( ) N5,000-10,000 ( ) Above N10,000 ( )

- Source of monthly allowance

Parents/guardian ( ) other family member ( ) intimate partner ( ) part time job ( ) other ( )

- Age of 1^st^ intercourse

Less than 16 years ( ) 16-20 years ( ) 21-24 years ( )

- Nature of 1^st^ intercourse

Consensual ( ) Not consensual ( )

Consensual sex means: actively agreeing to be sexual with someone and let them know sex is wanted.

**Knowledge and Awareness of Gender Based Violence (GBV) and Post-SBV health services:**

Gender based violence refers to violence that targets individuals or groups on the basis of their gender.

- GBV happens when someone experiences the following in an intimate partner relationship, tick all that applies:

Physical violence ( )

Sexual violence ( )

Emotional violence ( )

Economic violence ( )

- Preventing sexual-based violence helps to prevent exposure to HIV Yes( ) No ( ) Not sure ( )
- Sexual-based violence can lead to unintended pregnancies Yes ( ) No ( ) Not sure ( )
- Are you aware of the following:
- Contraceptives Yes( ) No ( ) Not sure ( )
- Emergency contraception Yes ( ) No ( ) Not sure ( )
- Condoms Yes ( ) No ( ) Not sure ( )
- Post-exposure prophylaxis for HIV Yes ( ) No ( ) Not sure ( )
- Have you ever used any of these?
- Contraceptives Yes( ) No ( ) Not sure ( )
- Emergency contraception Yes ( ) No ( ) Not sure ( )
- Condoms Yes ( ) No ( ) Not sure ( )
- Post-exposure prophylaxis for HIV Yes ( ) No ( ) Not sure ( )
- Have you ever been concerned or worried about your health and well-being while or after having sex with a partner? Yes ( ) No ( ) Not sure ( )
- If Yes, did you seek support or speak to anyone? Yes ( ) No ( ) Not sure ( )
- If Yes, did you seek support from a post-SBV health service facility? Yes ( ) No ( ) Not sure ( )

( Post-S*BV health services are health services that provide specialized care to people who have experienced a form of gender based violence*)

**Perception and Experiences of Post Sexual-Based Violence Health Services**

- A post sexual-based violence health service should be the first place to go following rape

Strongly agree ( ) Agree ( ) Neutral ( ) Disagree ( ) Strongly disagree ( )

- Health care workers are able to prevent sexual based violence from reoccurring

Strongly agree ( ) Agree ( ) Neutral ( ) Disagree ( ) Strongly disagree ( )

- Health care workers are available to offer post exposure prophylaxis for the prevention of HIV following rape

Strongly agree ( ) Agree ( ) Neutral ( ) Disagree ( ) Strongly disagree ( )

- Post sexual-based violence health services are effective to offer services for the prevention of pregnancy following rape

Strongly agree ( ) Agree ( ) Neutral ( ) Disagree ( ) Strongly disagree ( )

- Health care workers can offer or link psychological and counseling services to SBV survivors

Strongly agree ( ) Agree ( ) Neutral ( ) Disagree ( ) Strongly disagree ( )

- Health care workers can link SBV survivors to legal AID services

Strongly agree ( ) Agree ( ) Neutral ( ) Disagree ( ) Strongly disagree ( )

- Health care workers can be helpful in collection of evidence for forensic medical services

Strongly agree ( ) Agree ( ) Neutral ( ) Disagree ( ) Strongly disagree ( )

- SBV survivors should visit post-SBV health services to treat physical injuries

Strongly agree ( ) Agree ( ) Neutral ( ) Disagree ( ) Strongly disagree ( )

**Barriers in accessing Post SBV-health services**

On a scale of 1-7 (1 being most experienced barrier and 7 being the least experienced), rate the barriers that you think young women experience in accessing post-SBV health services.

- Difficulty in accessing post-SBV health services due to distance of post-SBV health service facility ( )
- Financial barriers (cost of accessing services) ( )
- Negative and unfriendly attitude, including distrust of health care providers ( )
- Lack of awareness of existing post-SBV health care services ( )
- Stigma ( )
- Shame ( )
- Lack of support systems in the home, school, and community to facilitate visit to Post-SBV health services ( )
- Belief that violence was normal ( )
- Fear of HIV testing ( )
- Fear of reporting the offender ( )

**Facilitators in accessing Post-SBV-health services**

On a scale of 1-9 (1 being most felt facilitator and 9 being the least felt facilitator), rate the facilitators in accessing post-SBV-health services (online or in-person services).

- Free/Affordable post-SBV health services ( )
- Support systems in homes, schools, and communities to facilitate access to post-SBV health services ( )
- Availability of NGOs/community organizations that can provide referral services to post-SBV health services
- Presence of post-SBV health service facilities in the community people live e.g. universities ( )
- Availability of post-SBV services as part of the larger general health care services ( )
- Effective care and support from post-SBV health services/health workers ( )
- Assurance of confidentiality after visiting a post-SBV health services ( )
- Accessing Post-SBV services without age limitations
- Availability of health care workers that will provide non-discriminatory post-SBV health services ( )

You have come to the END of the questionnaire.

***If you experience distress as a result of responding to this questionnaire, please contact Ms Amaka, Centre Coordinator, SOAR Child and Teen Support Centre on 08189606062.***

***Some other services available to access post-SBV services include;***

- *Mirabel Centre, Lagos state University Teaching Hospital, Ikeja, Lagos. Phone no: 08176275695, 08155770000*
- *FCT- Sexual and Gender Based Violence Response Team, Social Development Secretariat 2^nd^ Floor, Cyprian Ekwensi House, Arts and Culture Complex, Area 10 Garki, Abuja. Phone no: 08077111126.*
- *Other post-SGBV service centers in Nigeria can be found here: https://www.mediaconcern.net/sexual-assault-centers*

Thank You.
