# Supplementary material for: Barriers and facilitators to accessing post sexual-based violence health services among young women attending higher education institutions in Nigeria
Source: BMC Womens Health. 2025 Apr 19;25:193. doi: 10.1186/s12905-025-03714-2 (PMC12008884; doi:10.1186/s12905-025-03714-2)
Supplement: Supplementary file 2 — Supplementary Material 2 [file 12905_2025_3714_MOESM2_ESM.docx]

**Participant Information Sheet**

**Project Title**: Barriers and Facilitators in Accessing Gender-Based Violence (GBV) Health Services among Young Women (18-24 years) enrolled in Universities/Higher Institutions in Nigeria- A pilot online survey study.

You are invited to take part in a research study. To help you decide whether or not to take part, it is important for you to understand why the research is being done and what it will involve. Please take time to read the following information carefully. Talk to others about the study if you wish. Contact us if there is anything that is not clear, or if you would like more information. Take time to decide whether or not you wish to take part.

| **What is the purpose of the study?** |
| --- |
| I’m Ajoke Adebisi, a Master of Public Health student at the University of Edinburgh and this study is part of my research training. First it is important to note that a World Health Organisation study on violence and women’s health across 10 countries showed that 13-62% of women had experienced violence during their lifetime, with 29% reporting violence within the past year, and only 3% seeking Gender Based Violence (GBV) health services. The input of the health systems is vital in assisting women to reduce the impact of sexual and gender based violence (SGBV), and plays a role in preventing the occurrence of future violence but there is underutilization of GBV health services in sub-Saharan African countries.  This pilot study will be useful in exploring the barriers and facilitators in accessing GBV-health services among young women (18-24 years) in Nigeria. The study will add to the body of knowledge on the utilization of GBV-health services, provide data and evidence for improved public health programs and policies, and pilot/test the survey instrument. A convenience sample will be recruited in the time scale available, and young women 18-24 years attending Universities/Higher Institutions in Nigeria will be involved in the study.  NB: Gender based violence refers to violence that targets individuals or groups on the basis of their gender. |
| **Who can take part?** |
| We would like young women aged 18-24 years enrolled in a Nigerian University or Higher Institution as an undergraduate student to share with us their views about the barriers and facilitators to accessing gender-based violence (GBV) health services in Nigeria.  If you are a male student, a post-graduate student, below 18 years or above 24 years this study is not for you. |
| **Do I have to take part?** |
| No, it is up to you to decide whether or not to take part. You will be asked to answer the first question of the survey where you will give consent to take part in the study. If you decide to take part you are still free to withdraw at any time and without giving a reason. There is no penalty for refusing to take part in the study or for withdrawing from the study. |
| **What will happen if I take part?** |
| You have been directed to receive this information sheet and consent form via one of the social media platforms where the study has been advertised. Please take your time to read all of the information carefully before deciding whether or not to take part. If you consent to take part, you will be asked to complete the questionnaire which is available to view here xxx. The study will take about 10 minutes of your time; but the time you give to complete the questionnaire will be appreciated.  The first question of the questionnaire will ask you if you consent to take part in this study. If you click ‘yes’ and agree to take part, you will be directed to the main questionnaire to complete the questions, which will include questions on socio-demographic information. If you click ‘no’ and decline to take part, you will be directed to a thank you closing page. |
| **What are the possible benefits of taking part? What are the possible disadvantages of taking part?** |
| There may be no direct benefits to taking part in the study, but findings from the study will be helpful to add to the body of knowledge and inform public health programs and policies for an improvement in utilization of gender-based violence health services. The study is also part of the requirements for an MPH degree, and your support to my research training will be appreciated.  You are likely to take around 10 minutes to complete the questionnaire, but the time you spend completing the questionnaire will be appreciated. |
| **What will happen if I don’t want to carry on with the study** |
| You can withdraw from the study at any point before submission of the survey questionnaire by exiting the survey. A limitation is that if you complete and submit the questionnaire, it will be impossible to withdraw your information from the study because data will be anonymous at the point of submission, and it will not be possible for your responses to be traced. |
| **Will my taking part be kept confidential?** |
| All the information we collect during the course of the research will be kept private and there are strict laws which safeguard your privacy at every stage. |
| **What will happen to the results of the study?** |
| This study will be written up as an MPH Dissertation, and there are no intentions of making the study results available publicly for participants to see. However, the study may eventually be written up as a publication but you will not be identifiable from any published results. |
| **Who is organising and funding the research?** |
| This is a Masters level study which is being conducted by the researcher, Ajoke Adebisi, a Master of Public Health (MPH) student at the University of Edinburgh, Scotland, UK. The study is sponsored by the University of Edinburgh, Scotland, UK. |
| **Who has reviewed the study?** |
| The study proposal has been reviewed and approved by MPH Ethics Group at the Usher Institute, University of Edinburgh, and also by the Federal Capital Territory Administration of Nigeria. |
| **Researcher Contact Details** |
| If you have any further questions about the study please contact the researcher Ajoke Adebisi, on 08171963225 or email on: A.E.Adebisi@sms.ed.ac.uk. |
| **Supervisor Contact Details** |
| If you have concerns or questions you would like to ask the researcher’s Dissertation Supervisor please contact Dr Zhong Eric Chen on Eric.Chen@nhslothian.scot.nhs.uk |
| \| **Data Protection** \| \| --- \| \| All the information we collect during the course of this study will be kept confidential and there are strict laws which safeguard your privacy at every stage. We will ask for only the minimum personal information we need for the study.  **How I will use information about you?**  The researcher will need to collect the following personal identifiable information from you in order to conduct this research project:   - Age - Marital Status - Place of residence - Location of school - Level of parents/guardian education - Primary occupation of parents/guardian - Monthly allowance - Source of monthly allowance   The data collected from you from the questionnaire/survey will be uploaded to a password-protected Microsoft Excel spread sheet in order to conduct the analysis. Throughout this research study, active research data (including the personal data listed above) will be uploaded and stored on the University of Edinburgh’s One Drive network – a secure platform in line with the requirements of GDPR. The data collected from you will only be accessible to the researcher conducting this study.  Data collected (including personal data) will be retained on the University of Edinburgh One Drive secure platform for a period of 3 years and thereafter, the data will be permanently deleted.  **Who to contact if you experience distress as a result of responding to this questionnaire/survey** \|   ***If you experience distress as a result of responding to this questionnaire, please contact Ms Amaka, Centre Coordinator, SOAR Child and Teen Support Centre on 08189606062.***  ***Some other services available to access GBV health services include;***   - *Mirabel Centre, Lagos state University Teaching Hospital, Ikeja, Lagos. Phone no: 08176275695, 08155770000* - *FCT- Sexual and Gender Based Violence Response Team, Social Development Secretariat 2^nd^ Floor, Cyprian Ekwensi House, Arts and Culture Complex, Area 10 Garki, Abuja. Phone no: 08077111126.* - *More GBV service centers in Nigeria can be found here: https://www.mediaconcern.net/sexual-assault-centers*  \| **Privacy Notice** \| \| --- \|   The University of Edinburgh is the sponsor for this study based in the United Kingdom but this study is being conducted in Nigeria. The Sponsor has overall responsibility for the running of the study. To follow the United Kingdom’s data protection regulations, we must inform you of how we will use and store your personal data.  As a university, we use personally-identifiable information to conduct research to improve health, care and services. As a publicly-funded organisation, we have to ensure that it is in the public interest when we use personally-identifiable information from people who have agreed to take part in research. This means that when you agree to take part in a research study, we will use your data in the ways needed to conduct and analyse the research study.  We will use information from you in order to undertake this study. The sponsor will keep identifiable information about you for 3 years after the study has finished.  The University of Edinburgh will act as the data controller for this study. This means that they are responsible for looking after your information and using it properly.  Your rights to access, change or move your information are limited, as we need to manage your information in specific ways in order for the research to be reliable and accurate. If you withdraw from the study, we will keep the information about you that we have already obtained. To safeguard your rights, we will use the minimum personally-identifiable information possible.  Non-identifiable data from this project may be stored in a research data repository at the University of Edinburgh to allow knowledge sharing and learnings about this study. The University of Edinburgh provides its researchers (and their collaborators) two services for sharing and archiving of data which will be used for your information. There is an open access repository for anonymised data, which means that all non-identifiable data is freely available. For sensitive information a secure repository is used which can only be accessed by approved researchers who have undergone a rigorous application and review process.  **CONSENT FORM**  ***Please now read the consent points below:***   \|  \| \| --- \| \| 1. I confirm that I have read and understand the information sheet (25/11/ 2021 and Version 1.0) for the above study. I have had the opportunity to consider the information, ask questions and have had these questions answered satisfactorily. \| \| 1. I understand that my participation is voluntary and that I am free to withdraw at any time, without giving any reason and without any care or support and/or legal rights being affected. \| \| 1. I understand that the information I provide including personal data, will be kept confidential, stored securely and only accessed by those carrying out the study. I understand that relevant sections of my data collected during the study may be looked at by individuals from the Sponsor (University of Edinburgh), from regulatory authorities where it is relevant to my taking part in this research. I give permission for these individuals to have access to my data. \| \| 1. I give permission for my personal information to be passed to the University of Edinburgh for administration of the study. \| \| 1. I understand that my data collected in this study (including personal data) will be uploaded and transferred to the University of Edinburgh’s OneDrive secure network platform. I understand that this server is based in the United Kingdom, where the Data Protection laws may be different to those of my home country. \| \| 1. I understand that the results of this study will be written up in a dissertation and only the de-identified data may be used in future studies or future publications and presentations. I understand I will not be personally identified from any of the results of the study . \| \| 1. I agree to take part in the above study. \| |
